# Localised Grey Matter Atrophy in Multiple Sclerosis and Clinically Isolated Syndrome—A Coordinate-Based Meta-Analysis, Meta-Analysis of Networks, and Meta-Regression of Voxel-Based Morphometry Studies

**DOI:** 10.3390/brainsci10110798

**Published:** 2020-10-30

**Authors:** Sonika Singh, Christopher R. Tench, Radu Tanasescu, Cris S. Constantinescu

**Affiliations:** 1 Division of Clinical Neuroscience, Clinical Neurology, Queen’s Medical Centre, University of Nottingham, Nottingham NG7 2UH, UK; sonika.singh@nottingham.ac.uk (S.S.); radu.tanasescu@nottingha.ac.uk (R.T.); cris.constantinescu@nottingham.ac.uk (C.S.C.); 2 NIHR Nottingham Biomedical Research Centre, Queen’s Medical Centre, University of Nottingham, Nottingham NG7 2UH, UK; 3 Department of Neurology, Nottingham University Hospitals NHS Trust, Nottingham NG7 2UH, UK; 4 Department of Neurology, Division of Clinical Neurosciences, Colentina Hospital, University of Medicine and Pharmacy Carol Davila Bucharest, 020021 Bucharest, Romania

**Keywords:** coordinate-based meta-analysis, multiple sclerosis, clinically isolated syndrome, grey matter, atrophy

## Abstract

Background: Atrophy of grey matter (GM) is observed in the earliest stages of multiple sclerosis (MS) and is associated with cognitive decline and physical disability. Localised GM atrophy in MS can be explored and better understood using magnetic resonance imaging and voxel-based morphometry (VBM). However, results are difficult to interpret due to methodological differences between studies. Methods: Coordinate-based analysis is a way to find the reliably observable results across multiple independent VBM studies. This work uses coordinate-based meta-analysis, meta-analysis of networks, and meta-regression to summarise the evidence from voxel-based morphometry of regional GM hanges in patients with MS and clinically isolated syndrome (CIS), and whether these measured changes are relatable to clinical features. Results: Thirty-four published articles reporting forty-four independent experiments using VBM for the assessment of GM atrophy between MS or CIS patients and healthy controls were identified. Analysis identified eight clusters of consistent cross-study reporting of localised GM atrophy involving both cortical and subcortical regions. Meta-network analysis identified a network-like pattern indicating that GM loss occurs with some symmetry between hemispheres. Meta-regression analysis indicates a relationship between disease duration or age and the magnitude of reported statistical effect in some deep GM structures. Conclusions: These results suggest consistency in MRI-detectible regional GM loss across multiple MS studies, and the estimated effect sizes and symmetries can help design prospective studies to test specific hypotheses.

## 1. Introduction

Areas of inflammation, axonal loss, demyelination and gliosis, occurring throughout the brain and spinal cord, are the distinctive features of multiple sclerosis (MS) [1]. Although MS has been considered a condition affecting the white matter (WM) and the hyperintense lesions on T2-weighted images are for MS diagnostics, there is a limited association between lesion accrual and disability. Atrophy measures appear to be a more specific marker of MS pathology than lesion volumes [2], as demonstrated by the association of atrophy in the brain and spinal cord with increasing disability [3]. In addition, progressive ventricular enlargement, another indicator of atrophy, has been shown to predate clinically definite MS in patients with clinically isolated syndrome (CIS) [4]. 

Atrophy of the grey matter (GM) is already observed in the initial disease stages [5] and an association has been observed with cognitive decline and physical disability [6]. The underlying mechanism for GM atrophy is unknown, but several hypotheses have been postulated including primary GM damage involving neuronal loss, demyelination, reduced synapses, decreased oligodendrocytes and axonal transection [7]. An association has been demonstrated between GM loss and lesion load even in patients with short disease duration [8,9,10,11]. 

The importance of GM loss in MS necessitates careful analysis using advanced imaging methods such as voxel-based morphometry (VBM). Multiple VBM analyses of MS or CIS patients compared to healthy control groups have been published and significant changes interpreted as atrophy. VBM has been shown to be robust against various processing steps with false positives randomly distributed about the brain [12] However, studies often involve small sample sizes, and with lack of power comes increased chance that any observed effect is a false positive [13]. Moreover, uncorrected p-values are commonly employed, inflating the false-positive rates [14]. A complexity of VBM was highlighted by a study [15] that compared detectable GM changes found using different software packages: FSL [16], FreeSurfer [17,18], and SPM (Statistical Parametric Mapping Functional Imaging Laboratory, University College London, London, UK). The study examined agreement between these packages by using an MS cohort with a common disease type with matched controls and highlighted pronounced differences. 

Given the problems with single studies, there is potential for meta-analyses to reveal which of the observed effects are most likely to indicate MS-specific GM changes. Results can add to the understanding of GM pathology in MS, and provide specific hypotheses for testing. In the absence of the original images, a coordinate-based meta-analysis (CBMA) is possible using only the summary reports tabulated in the large majority of VBM publications. Results indicate effects most reliably detectable by VBM. 

The primary aim of this meta-analysis was to determine the locations of consistent regional GM changes in MS and CIS patients by means of a coordinate-based random effect size (CBRES) [19] meta-analysis and coordinate-based meta-analysis of networks (CBMAN) [20]. Each of these algorithms cluster the reported coordinates where there is spatial concordance. CBRES performs conventional random effect meta-analysis, of the reported Z scores standardised by study sample size, in each cluster. CBMAN looks for network-like patterns of GM loss by considering significant correlations of standardised statistical effects between pairwise clusters. Secondary analyses involving subgroup analysis and meta-regression are also performed using CBRES. The coordinate data used in this analysis is made available on the Nottingham Research Data Management Repository [dataset] (DOI: 10.17639/nott.7049) for validation purposes [21]. 

## 2. Materials and Methods

### 2.1. Search Strategies

A literature search was conducted using PubMed, with the following search term combinations: (“multiple sclerosis” [All Fields] OR “ms” [All Fields] OR CIS [All Fields] OR “clinically isolated syndrome” [All Fields]) AND (“voxel based morphometry” [All Fields] OR VBM [All Fields]) AND (“atrophy” [MeSH Terms] OR “atrophy” [All Fields]) AND (“grey matter” [All Fields] OR “gray matter” [All Fields] OR GM[All Fields]); Web of science, using the following search terms: TS = (“multiple sclerosis” OR MS OR CIS OR “clinically isolated syndrome”) AND TS = (“voxel based morphometry” OR VBM) AND TS = (atrophy) AND TS = (“grey matter” OR “gray matter” OR GM); and Science direct, using the following search terms: TITLE-ABSTR-KEY(“multiple sclerosis” OR MS OR “clinically isolated syndrome” OR CIS) and TITLE-ABSTR-KEY(“voxel based morphometry” OR VBM) and TITLE-ABSTR-KEY(“grey matter” OR “gray matter” OR GM) and TITLE-ABSTR-KEY(atrophy). 

### 2.2. Study Selection

Inclusion criteria are (a) involved participants with MS or CIS, (b) compared patients to healthy controls, (c) performed whole-brain VBM for assessing GM atrophy, and (d) reported coordinates for GM volume changes in either Talairach [22] or Montreal Neurological Institute (MNI) reference space. Exclusions were made due to unreported coordinates or unavailable full text. Two independent researchers assessed these criteria of the individual studies and the MNI or Talairach coordinates. 

### 2.3. Study Properties

Information extracted for analysis: the censoring threshold, i.e., the smallest Z value the study considered as significant, the reported coordinates, and either the Z score, estimated degrees of freedom and t statistic, or uncorrected p-value; t statistics and uncorrected p-values are converted automatically to Z scores. 

### 2.4. Coordinate-Based Meta-Analysis

All CBRES and CBMAN analyses are performed using NeuRoi (https://www.nottingham.ac.uk/research/groups/clinicalneurology/neuroi.aspx), which is available to use freely.

Details about the algorithms incorporated into CBRES and CBMAN are presented in [19,20]. In both algorithms a clustering algorithm [23] is used to determine where the coordinates reported by multiple independent studies are spatially concordant (clustered). Once clusters are formed, the reported Z scores are converted to standardised effect sizes by dividing by the square root of the number of subjects. In CBRES, a random effect meta-analysis of these effect sizes is performed in each cluster. In CBMAN, the test statistic is the correlation of standardised effect sizes performed pairwise between clusters. Where a study does not report a coordinate within a cluster, or where no effect sizes are reported by a study, the contribution to the cluster is estimated using the study censoring threshold.

The significant results of the CBRES meta-analysis are clusters of reported coordinates where the estimated effect size is statistically different to zero after controlling the false cluster discovery rate (FCDR), a type 1 error control method based on the false discovery rate (FDR) [24]. The expected proportion of clusters incorrectly declared significant is controlled at 5% by default. Clusters indicate both spatial and effect size concordance across studies, which is an unlikely chance event suggesting that atrophy at the location of the clusters is a general feature of MS. 

Significant results reported by CBMAN are clusters where standardised reported statistical effects are correlated between clusters. This indicates a significant pattern of reported effect that is represented as a network of nodes (clusters) and edges (correlations). The FDR is used to control type 1 error rate of the effect size correlations. The clusters analysed by CBMAN and CBRES are identical, since the same clustering algorithm is employed, but the results may differ due to the different hypotheses tested. A feature of both CBRES and CBMAN is that the results declared significant are reported as a function of the FDR. Any that just miss the threshold for significance can therefore be explored.

Analysis can also be performed on subgroups of studies. This estimates a subgroup-specific effect size in each of the clusters found significant during the full analysis (using all studies); this is useful since clusters may not be significant if the subgroup is small, yet the effect size might be of interest. Furthermore, the use of standardised effect sizes makes meta-regression possible by looking for significant correlation between a specified covariate and the standardised effect size in each cluster. 

### 2.5. Experimental Procedure

Multiple experiments reported on the same subjects were pooled into single independent experiments to prevent correlated results inducing apparent concordance that is not due to a generalisable MS process [25]. 

All planned analyses were performed controlling the FDR at 0.05. For each, the next most significant clusters were explored, and reported, to make sure that none had just been missed at this threshold. 

### 2.6. Main Analysis

The main meta-analysis was performed using both CBRES and CBMAN and involved all studies meeting the inclusion criteria. 

### 2.7. Subanalyses

Subanalyses for CIS, benign MS (BMS), Relapsing Remitting MS (RRMS), Primary Progressive MS (PPMS) and Secondary Progressive MS (SPMS) studies were performed, with subtypes as defined in the reporting studies. This analysis estimates effects of the respective subgroup within significant clusters discovered using all studies.

### 2.8. Meta-Regression

Regression analyses were performed for covariates that might influence the grey matter volume: mean age (years), MS disease duration (years; excluding CIS studies with no MS disease duration), MSFC, and EDSS (all studies and including RRMS studies only).

## 3. Results

### 3.1. Included Studies and Sample Characteristics

**Figure 1:**
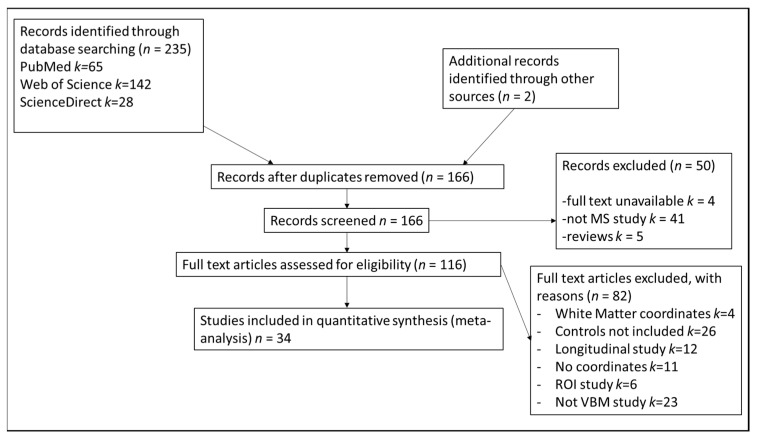
This is a figure with the PRISMA flowchart [26] showing the inclusion and reasons for exclusion of studies in the meta-analysis.

The literature search yielded 237 potential studies, of which 34 met the inclusion criteria (Figure 1). The 34 included research papers reported 45 whole-brain VBM experiments comparing MS subtypes and controls (see Appendix A for study details).

Studies included in the analysis were conducted between 2006 and 2018 and involved a total of 1615 patients and 1098 controls. The studies by MS subtype were 4 CIS, 24 RRMS, 7 PPMS, 3 BMS, and 2 SPMS; 4 studies were not specific to any single MS subtype. The mean patient age was 40.56 years (*SD* = 5.76). The mean (standard deviation (*SD*)) EDSS was 2.7 (1.5). The mean disease duration was 9.26 (6.51) years. Across the studies the duration of disease, excluding the CIS studies, was 1.66 to 30.50 years.

### 3.2. Primary Meta Analysis

**Figure 2:**
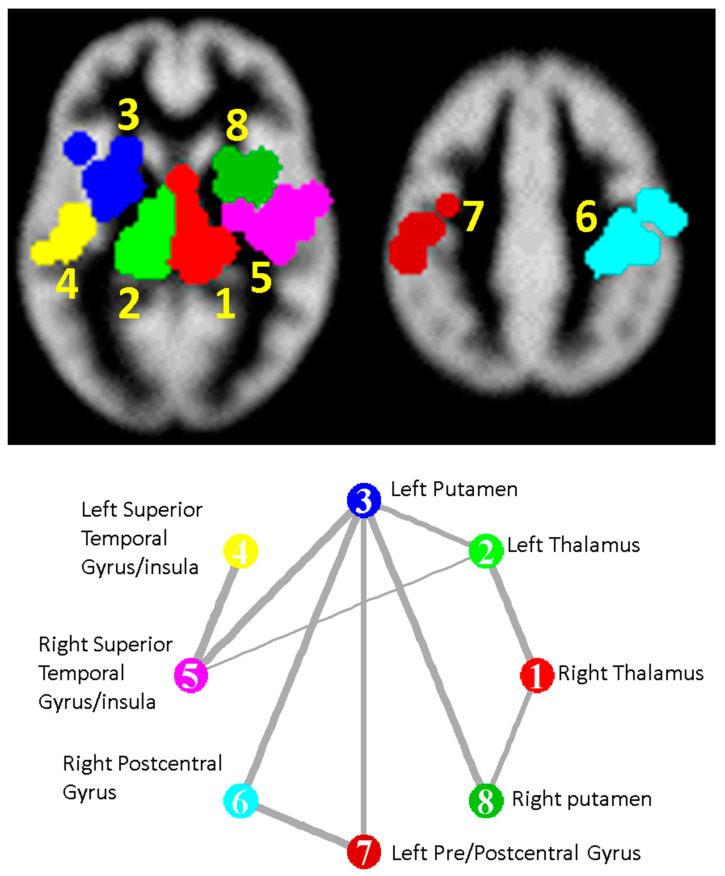
Top: Significant clusters of GM atrophy detected using the CBRES and CBMAN algorithms. Cluster 7 is detected only by CBMAN. Bottom: The network edges found to connect the clusters significantly by CBMAN; line thickness indicates correlation strength of the standardised effect sizes between connected clusters.

**Figure 3:**
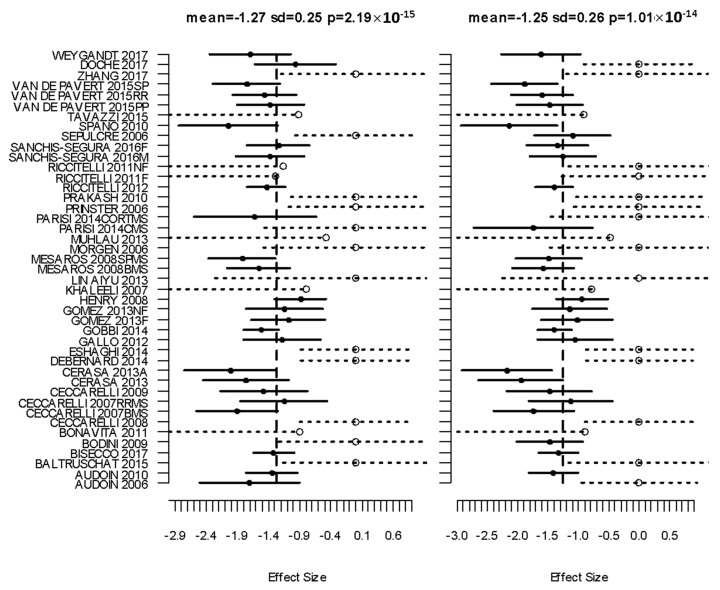
Forest plots for the two most significant clusters (left and right Thalamus) of GM atrophy reported by the 45 VBM experiments; forest plots for the other significant clusters are shown in Appendix B. Markers with a solid circle indicate the effect size reported by the study in the respective cluster. The solid horizontal lines span ± 1.96 times the within-study standard deviation of the effect size. Censored values are depicted by open circle markers and the intervals by dashed lines (⋅⋅⋅o⋅⋅⋅).

**Figure 4:**
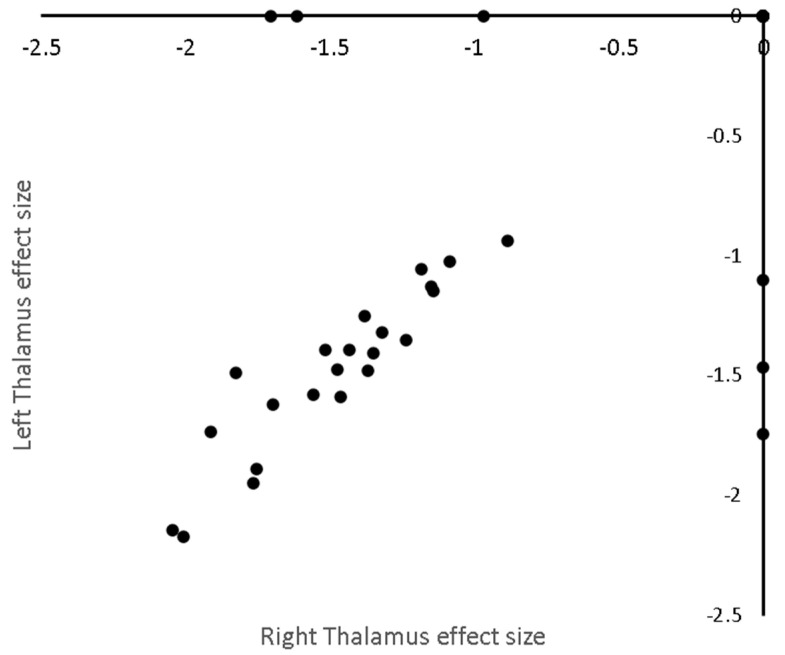
Relationship between standardised effect sizes reported in the left and right thalamic clusters. Markers that fall on the axes are censored.

The analysis found eight significant clusters involving basal ganglia and cortical regions; effect sizes are given in Table 1 and the complete list of Talairach regions, automatically detected [26], covered by each cluster given in online materials [dataset] (DOI: 10.17639/nott.7049) [21]. Significant clusters and a depiction of the covariance of standardised effect sizes between clusters are shown in Figure 2. Forest plots for the most significant clusters according to CBRES are shown in Figure 3. In Figure 4, a scatter plot of standardised effect sizes reported in the left and right thalamic clusters shows clear correlation detected by CBMAN. The first non-significant cluster according to CBRES was at a FCDR of 0.18. The first non-significant edges discovered by CBMAN were at a FDR of 0.057, where a further nine significant edges and two extra clusters (right Caudate peaking at Talairach coordinates {12, 4, 20} mm and another covering mostly the left/right cingulate gyrus peaking at {−4, −18, 44} mm) are found.

### 3.3. Subanalyses

Results from the subanalysis are given in Table 1, which shows estimated effects sizes considering only the respective subgroup within each of the clusters from the primary meta-analysis. It is apparent that the estimated effects are lowest in magnitude in the CIS and PPMS groups and highest in the SPMS and BMS groups, while RRMS generally falls in the middle.

### 3.4. Metaregresion

#### 3.4.1. Age

A single cluster was found with age as a significant covariate, as shown in Table 2. The first non-significant cluster was at a FCDR of 0.18.

#### 3.4.2. MS Disease Duration

This analysis included 39 of the independent experiments reported MS disease duration due to exclusion of CIS studies where MS disease duration is zero. Three clusters were found with disease duration as a significant covariate, as shown in Table 2. The first non-significant cluster was found at a FCDR of 0.1.

#### 3.4.3. MSFC

Regression analysis was not performed because only 9 out of 44 studies reported MSFC.

#### 3.4.4. EDSS

No significant clusters with EDSS as a covariate. The first non-significant cluster was found at a FCDR of 0.1.

## 4. Discussion

The results of 34 voxel-based morphometry studies of MS and CIS are summarised using CBMA, showing that GM atrophy in MS not only occurs in some regions more than in others and that regions of predilection are not independent. Eight regions were identified using two algorithms testing different null hypotheses. Results indicate a consistent pattern, rather than independent clusters, of reported effects both spatially and in terms of effect size.

The pattern of localised GM atrophy involves both cortical and subcortical regions. Considering the correlation of reported statistical effect size suggests that these regions do not develop independently, but rather together and with some hemispheric symmetry as shown in Figure 2. Meta-regression analysis suggests that the standardised effect magnitude increases with disease duration by several % per year of disease on average. This is also reflected in the mean statistical effect size estimates within the disease type subgroups, where the estimates for the CIS group tend to be lower than the RRMS group, which are in turn lower than the SPMS group. The PPMS subgroup reported intriguingly low statistical effect sizes, while the BMS group almost as large as the SPMS group in some clusters, which might reflect that BMS is indistinct from MS with long enough follow up. These estimates should be considered with caution because of the small subgroup sizes, however they could be prospectively tested.

GM tissue damage is an important pathological process in MS that underlies neurological disability [27]. It has been suggested that distribution of cortical GM atrophy is related to the effects of WM lesions on cortical regions that are network hubs, with trans-synaptic degeneration then extending from these hubs [28], or that the preferential accumulation of WM lesions in some regions would induce tract-mediated effects through secondary retro- or anterograde degeneration. The relationship between GM atrophy and WM abnormalities is weaker in people with PPMS or SPMS [29]. The loss of volume is the result of many dynamic processes, with a balance between destructive and reparative mechanisms with interaction among neurons, oligodendrocytes, axons, microglia, astrocytes, inflammatory cells, endothelial cells and water distribution [30]. 

In the thalamic clusters, the standardised effect sizes were found to correlate negatively with disease duration. Both imaging and pathology studies have demonstrated the involvement of thalamus in early RRMS [31], CIS [32] and paediatric MS [33]. Cifelli and colleagues [34] conducted a study of normalised thalamic volume measurements in SPMS patients. Volumes of manually outlined thalami were normalised by intracranial volumes and showed a mean decrease of 17%. Thalamic volume loss may be due in part to disconnection created by WM lesions [35,36]. 

Atrophy of the left and right putamen MS has also been detected. The putamen is part of the dorsal striatum and the basal ganglia and plays a role in the regulation of movement, coordination, motor function and cognition [37,38,39]. It is also involved in modulation of sensory and motor aspects of pain [40]. Thus, a pathology such as neurodegeneration might be expected to cause a broad spectrum of clinical manifestation from motor dysfunction to psychiatric disorder [41,42]. Previous studies have demonstrated progressive atrophy of the putamen in both RRMS and SPMS [43]. Kramer and colleagues [44] recently reported a significant relationship between putamen volume and disease duration in MS, which was also indicated by the present study.

Pre- and postcentral gyrus (bilateral) is consistently reported. The clusters have density peaks reported in the right precentral and the left postcentral gyrus, but the coordinates forming the clusters cover both pre- and postcentral gyrus on each side. Li et al. [45] used diffusion tensor imaging (DTI) and demonstrated neuroconnectivity changes in the left postcentral gyrus, and reduced communicability correlating with the 25 foot walk test results. 

Clusters covering the left/right superior temporal gyrus and insula ware also detected. The superior temporal gyrus is associated with auditory and speech comprehension [46,47] and perception of emotions in facial stimuli [48,49]. In addition, it is an essential structure in the pathway containing prefrontal cortex and amygdala that are responsible for social cognition processes [48,50]. The study conducted by Achiron and colleagues [51] suggested correlation between reduced cortical thickness in superior temporal gyrus and global cognitive score, attention, information processing speed and motor skills. The insula is primarily a visceral-somatic region [52]. Studies have shown a relationship between functional connections of the basal ganglia and insula and fatigue severity in case of MS patients [53,54,55]. 

A similar coordinate-based meta-analysis in MS was performed by Chiang et al. at the same time as the present study [56]. That study used the popular ALE algorithm and produced similar results. The study also used functional meta-analytic connectivity modelling (fMACM) [57] to explore functional coactivation of clusters as a network, estimated using non-MS studies. By contrast, the present study investigates the network-like properties of GM atrophy and uses the included MS-specific studies. 

The limitations of CBMA are that bias and methodological issues in the primary studies might be reflected in the results. Therefore, CBMA results should be considered hypothesis generating and used to inform robust prospective studies. To this end, the presented results provide a priori regions of interest for testing as well as statistical effect size estimation for sample size calculations.

## 5. Conclusions

This CBMA of VBM studies of MS and CIS has identified a pattern of related cortical and subcortical GM atrophy. Relationships are indicated by the covariance of reported statistical effects. Disease duration was found to be a significant covariate of the standardised reported effect sizes in the thalamic clusters and a cluster covering the left claustrum/putamen/insula. The estimated statistical effect sizes may be important for powering prospective studies of GM atrophy in MS to test specific hypotheses. 

## Figures and Tables

**Table 1:** Shows significant clusters detected by the CBRES and CBMAN algorithms for the main meta-analysis and estimated effects from the subanalyses. The column ‘main analysis’ shows effect size, standard deviation and false cluster discovery rate for each significant cluster estimated using CBRES. The subsequent columns show the estimated effect size for the subanalyses; - signifies no contribution of the subgroup to the cluster. * Cluster 7 is discovered by the CBMAN algorithm only. [FCDR- False Cluster Discovery Rate; CIS- Clinically Isolated Syndrome; BMS- Benign MS; RRMS- Relapsing Remitting MS; PPMS- Primary Progressive MS; SPMS- Secondary Progressive MS; FCDR in bold—statistically significant]

Cluster Number	Talairach Labels	Talairach Coordinate of Density Peak (x,y,z)mm	Main Analysis Mean (*SD*); FCDR	CIS Subanalysis Mean	BMS Subanalysis Mean	RRMS Subanalysis Mean	PPMS Subanalysis Mean	SPMS Subanalysis Mean
1	Right Thalamus	(12.0 −28.0 8.0)	−1.27 (0.25); **0.00025**	−1.01	−1.76	−1.23	−0.94	−1.80
2	Left Thalamus	(−14.0 −28.0 8.0)	−1.25 (0.26); **0.00025**	−1.04	−1.74	−1.11	−1.25	−1.70
3	Left Putamen	(−28.0 2.0 6.0)	−0.96 (0.24); **0.00033**	−0.96	−1.28	−0.81	−0.96	−1.52
4	Left Superior Temporal Gyrus/Insula	(−48.0 −18.0 2.0)	−0.85 (0.19); **0.007**	−0.66	-	−0.85	−0.77	−1.09
5	Right Superior Temporal Gyrus/Insula	(38.0 −18.0 12.0)	−0.84 (0.2); **0.009**	-	-	−0.83	−0.55	−1.22
6	Right Postcentral Gyrus	(36.0 −26.0 48.0)	−0.87 (0); **0.0014**	−0.67	−1.18	−0.90	−0.78	−1.20
7	Left Pre- and Postcentral Gyrus *	(−46.0 −18.0 38.0)	−0.69 (0.3); **0.18**	−0.69	−1.03	−0.82	−0.79	−1.57
8	Right Putamen	(26.0 4.0 8.0)	−0.8 (0.31); **0.03**	-	−1.25	−0.69	−0.48	−1.27

**Table 2:** Significant clusters for the age and disease duration meta-regression. The location indicates the most commonly reported Talairach labels covered by the cluster.

Location	Talairach Coordinates (x,y,z) mm	Change in Standardised Effect per Year (% of Mean Effect)
Age		
Left Thalamus	(−14.3 −28.3 10.4)	−0.025 (2%)
Disease Duration		
Right Thalamus	(10.0 −30.0 7.0)	−0.049 (3.7%)
Left Thalamus	(−14.3 −25.2 6.1)	−0.046 (3.8%)
Left Claustrum, Putamen, and Insula	(−31.0 0.3 8.8)	−0.032 (3.4%)

**Figure A1:**
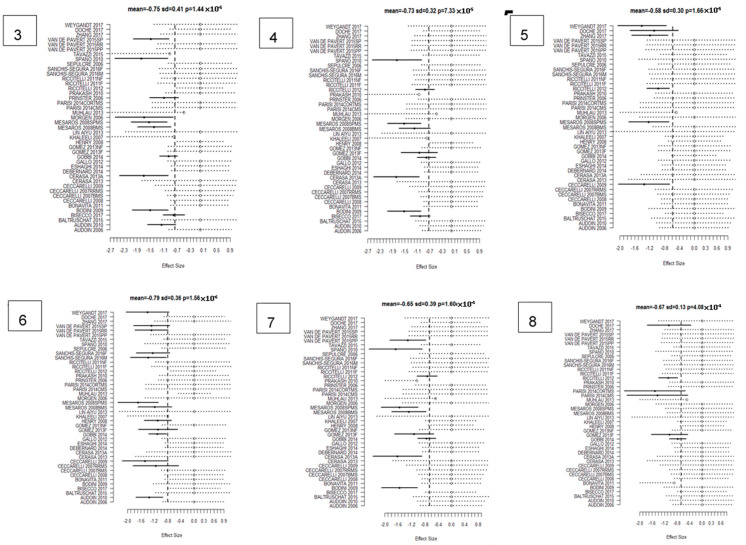
Forest plots for significant clusters 3–8.

## Appendix A

**Table A1:** Demographics for the included studies.

Study	Controls				Patients				
	*n*	Mean Age	*SD*/Range	Females	Subtype	*n*	Mean Age	*SD*/Range	Females
AUDOIN 2006	10	37	31–52	4	EARLY RRMS	21	36	27–55	16
AUDOIN 2010	37	28	8	-	CIS	62	29	20–46	-
BALTRUSCHAT 2015	15	30.47	5.91	7	RRMS	17	32.82	6.41	11
BISECCO 2017	52	37.3	13.1	33	RRMS	125	36.8	10.7	82
BODINI 2009	23	35.1	7.9	12	EARLY PPMS	36	44.8	11.13	15
BONAVITA 2011	18	39	10	10	RRMS	36	CI-40.9	8.7	11
	-	-	-	-			CP-40.5	6.9	10
CECCARELLI 2008	21	40.9	24–62	14	CIS	28	30.7	21–43	15
CECCARELLI 2007BMS	20	36.8	6.8	13	BMS	19	41.5	5.6	15
CECCARELLI 2007RRMS	-	-	-	-	RRMS	15	33.3	7.8	12
CECCARELLI 2009	17	51.3	26–68	11		18	49.6	38–73	10
CERASA 2013	20	36.9	5.8	14	RRMSnc	14	38.6	8.5	11
CERASA 2013A	-	-	-	-	RRMSc	12	38.9	8.7	10
DEBERNARD 2014	25	35.2	10.3	17	EARLY RRMS	25	37.2	8.6	22
ESHAGHI 2014	19	37.6	34.4, 41.9	9	PPMS	36	42.8	39.4, 46.6	12
GALLO 2012	15	36.3	20–53	10	RRMS	30	35.9	19–51	20
GOBBI 2014	90	39.7	13.7	51	MIXED_MS	123	41.7	10.3	71
GOMEZ 2013F	18	31.06	5.67	8	RRMS_fatigue	32	37.72	5.9	21
GOMEZ 2013NF	18	-	-	-	RRMS_nonfatigue	28	34.96	5.87	18
HENRY 2008	49	38	11	34	CIS	41	37	10	29
KHALEELI 2007	23	35.1	23–56	12	EARLY PPMS	46	43.5	19–65	19
LIN AIYU 2013	11	39.5	13.2	7	RRMS	11	38.5	12.2	7
MESAROS 2008BMS	21	45.7	25–66	11	BMS	60	46.2	35–63	37
MESAROS 2008SPMS	21	45.7	25–66	-	SPMS	35	46.5	30–63	25
MORGEN 2006	19	31.7	7.5	-	RRMS	19	33.05	8.26	-
MUHLAU 2013	49	36.4	13	33	CIS or RRMS	249	36.8	10.7	62
MORGE low PASAT	19	31.7	7.5	-	RRMS	10	36.7	8.05	-
PARISI 2014CMS	9	54.4	12.1	6	CMS	9	50.2	11	7
PARISI 2014CORTMS	9		-	-	CORT-MS	9	48.9	9.9	7
PRINSTER 2006	34	43.2	13.2	15	RRMS	51	38.6	7.5	36
PRAKASH 2010	15	45.8	1.8	15	RRMS	21	44.2	1.9	21
RICCITELLI 2012	88	39.7	18–65	51	RRMS	78	40.2	20–63	55
RICCITELLI 2011F	14	38.7	8.4	8	RRMS Fatigue	10	38	7.7	6
RICCITELLI 2011NF	14	-	-	-	RRMS nonfatigue	14	38.6	8.5	8
SANCHIS-SEGURA 2016M	35	25.54	5.35	-	RRMS male	22	38.68	8.72	-
SANCHIS-SEGURA 2016F	28	27.96	7.85	-	RRMS female	34	40.85	10.18	-
SEPULCRE 2006	15	43.2	10.9	6	PPMS	31	43.7	9.87	13
SPANO 2010	20	40.5	11.07	12	BMS	10	44.5	6.5	8
TAVAZZI 2015	31	47.9	14.5	20	PPMS	18	46.9	8.1	6
VAN DE PAVERT 2015PP	30	37.8	11.8	18	PPMS	25	52.5	9.8	14
VAN DE PAVERT 2015RR	30	-	-	-	RRMS	30	42.5	9.6	20
VAN DE PAVERT 2015SP	30	-	-	-	SPMS	25	52.8	7.6	14
ZHANG 2017	29	37.79	10.29	17	RRMS	39	38.26	9.05	23
DOCHE 2017	16	37.1	10.2	12	RRMS	23	34.2	9.3	19
WEYGANDT 2017	21	49.1	11.7	13	HI-LB MS	18	49.8	7.7	10

**Table A2:** Clinical characteristics of patients in the included studies.

	Disease Duration (*y*)	*SD*/Range	MSFC	EDSS	*SD*/Range	PASAT 3′	*SD*/Range	Education	*SD*/Range	BPF (mm^3^)	*SD*/Range
AUDOIN 2006	2.15	1.2–3.8	−0.348	1	0–3	-	-	-	-	-	-
AUDOIN 2010	0.33	0–0.5		1	0–3.5	40	10	13	3	0.831	0.043
BALTRUSCHAT 2015	4.53	3.5	-	2.24	1.09	48.12	6.94	12	2.72	0.84	0.023
BISECCO 2017	9.6	8.7	-	2	0–6	-	-	12.9	3.7	-	-
BODINI 2009	3.3	0.9	-	4.5	1.5–7	47.65	11.24	-	-	-	-
BONAVITA 2011	11.86	7.08	-	2.8	1.1	-	-	12.5	3.9	0.82	0.03
	10.91	4.67	-	2.6	1.7	-	-	12.3	3.6	0.83	0.03
CECCARELLI 2008	0	0	-	0	0–1	-	-	-	-	-	-
CECCARELLI 2007BMS	20	15–30	-	2	1.0–3.0	-	-	-	-	-	-
CECCARELLI 2007RRMS	6	2.0–10.0	-	1.5	1.0–3.5	-	-	-	-	-	-
CECCARELLI 2009	10.7	4.0–21.0	-	5.5	3.0–7.0	-	-	-	-	-	-
CERASA 2013	8.8	4.4	-	2	1.5–4.5	-	-	13	5.0–17.0	-	-
CERASA 2013A	12.1	8.7	-	2.5	1.0–4.0	-	-	13	5.0–17.0	-	-
DEBERNARD 2014	2.4	1.5	0.4 (0.6)	1.5	0–4.5	0.16	0.99	13.5	2.7	-	-
ESHAGHI 2014	3.3	2.9,3.6	−1.2 (−0.7, −1.6)	4	1.5, 7	-	-	-	-	-	-
GALLO 2012	9.2	3.0–22	-	2.1	1.0–5.5	-	-	-	-	-	-
GOBBI 2014	12.6	1.0–44	-	2	0–7.0	36.6	1.0–59	-	-	-	-
GOMEZ 2013F	7.44	5.15	-	3.2	1.68	-	-	-	-	-	-
GOMEZ 2013NF	5.14	3.69	-	1.96	1.2	-	-	-	-	-	-
HENRY 2008	0.3	0.25	1.9 (1.7)	1.1	0.8	−2.1	2.3	-	-	*n* GMV = 940(52)	-
KHALEELI 2007	3.3	2.0–5.0	-	4.5	1.5–7	-	-	-	-	-	-
LIN AIYU 2013	30.5	11.1	-	3.4	2.3	-	-	12.3	4.7	-	-
MESAROS 2008BMS	22.7	15–40	-	1.5	0–3.0	-	-	-	-	-	-
MESAROS 2008SPMS	16.2	7.0–27	-	6	4.0–7.0	-	-	-	-	-	-
MORGEN 2006	1.66	1.43	-	1	0–3.5	31	22–56	15 (college level)	-		-
MUHLAU 2013	-	-									
MORGE low PASAT	2.02	1.78	-	2	0–3.5	27	22–31	15 (college level)	-		-
PARISI 2014CMS	14	4–20	-	3	1.5–6	-	-	10.6 2.9		765 ml	31 ml
PARISI 2014CORTMS	8	2–31	-	4	1.0–6.0	-	-	8.4 3.2		655 ml	110 ml
PRINSTER 2006	13.1	6.4	-	2.6	1.5–4.5	-	-	-		-	-
PRAKASH 2010	7.3	0.1	-	2.2	0–6	43	2.3	15.6 0.4		-	-
RICCITELLI 2012	10	1–28	-	1.5	1–4.5	-	-	-		-	-
RICCITELLI 2011F	8.2	6.2		1.5	1.5–2.0	-	-	-		-	-
RICCITELLI 2011NF	10.6	6.6	-	1.5	0–1.5	-	-	-		-	-
SANCHIS-SEGURA 2016M	6.45	5.53		2.5	0–6.5	35.23	20.01	5 1–6		0.83	0.33
SANCHIS-SEGURA 2016F	8.82	7.47		2.38	0−6.5	33.35	23.04	4 1–6		0.83	0.27
SEPULCRE 2006	3	2–5	−0.26(−6. 16–0.79)	4.5	3.5–7	−0.002	−3.73–1.24	-		-	-
SPANO 2010	17.1	4.5	not for all pts	1.75	1–3	-	-	-		-	-
TAVAZZI 2015	12.4	7.73	-	6	3.0–8.0	-	-	-		-	-
VAN DE PAVERT 2015PP	12	7.4	−0.62 (0.81)	6	0–6.5	−0.7	1.38	-		-	-
VAN DE PAVERT 2015RR	11.5	10.5	−0.41 (0.76)	1.75	1.0–6.5	−0.69	1.32	-		-	-
VAN DE PAVERT 2015SP	24	8.2	−0.77 (0.66)	6.5	4.5–8.5	−0.94	1.12	-		-	-
ZHANG 2017	7.69	5.96	-	2.24	1.58	CI n CP separate values		11.9 3.68		-	-
DOCHE 2017	4.5	4.6	−0.70 (1.04)	1.5	1.2	-		-	-	-	-
WEYGANDT 2017	11.7	7.2	-	4	2.5–6.0	-		-	11	GM fraction = 0.41	0.04
